# Checklist and distribution of Collembola from Greater Puerto Rico

**DOI:** 10.3897/BDJ.8.e52054

**Published:** 2020-07-09

**Authors:** Claudia Marcela Ospina-Sánchez, Felipe N Soto-Adames, Grizelle González

**Affiliations:** 1 USDA-FS, International Institute of Tropical Forestry, San Juan, Puerto Rico USDA-FS, International Institute of Tropical Forestry San Juan Puerto Rico; 2 Florida Department of Agriculture, Tallahassee, FL, United States of America Florida Department of Agriculture Tallahassee, FL United States of America

**Keywords:** Caribbean, distribution, database, historical data, literature, Puerto Rico, springtails, Tropical Island.

## Abstract

**Background:**

Springtails (Arthropoda, Hexapoda, Collembola) are small arthropods commonly found in soil, litter and other habitats all around the Globe. More than 9,000 species have been described worldwide, but knowledge about their diversity and distribution remains far from complete. Reports of springtail diversity in the Antilles are uneven, some islands are relatively well known, whereas others have not been explored at all. The fauna of Puerto Rico is reasonably well known, but many recent reports are scattered in published literature and unpublished theses.

**New information:**

Here, we present a summary of all springtail species identified from the Bank of Puerto Rico, including unpublished records. As a result, we list 146 species including 43 unnamed, included in 65 genera and 17 families. Most species, 33, belong to Entomobryidae, but this possibly reflects the taxonomic expertise of specialists working in Puerto Rico rather than a real bias in the distribution of higher taxa in the islands. In addition to the new records, the database provides information on the world and local distribution of species listed. The dataset, presented here, is work in progress and will be updated as ongoing taxonomic inventories are completed.

## Introduction

The present checklist synthesises the occurrence and distribution of the springtail fauna of Greater Puerto Rico (i.e. Puerto Rico, Vieques, Culebra, Mona) and also makes and updates the available online dataset to the general scientific community through https://doi.org/10.2737/RDS-2018-0063 (Ospina-Sanchéz et al. 2019).

Puerto Rico is an archipelago located in the north-eastern part of the Caribbean and has an area of approximately 8900 km^2^ in land. The main island accounts for approximately 8746 km^2^ of that area, whereas the islands of Vieques and Culebra account for 131 km^2^ and 26 km^2^, respectively. In the main island, the altitude varies between sea level and 1300 m a.s.l. Temperatures tend to decrease towards higher elevations along the centre of the main island (Ewel and Whitmore 1973) and the mean annual temperature ranges from 19 to 26°C. The ecological life zones of Puerto Rico include subtropical dry forests and subtropical rainforests, amongst other life zones described by Ewel and Whitmore (1973). In addition, the island can be divided into three major physiographic regions, based on topographic features, geologic structure and rock type: the coastal lowlands, the coastal hills and the central mountains (Helmer et al. 2002, Parés-Ramos et al. 2008).

The first report of Collembola in Puerto Rico was made by Folsom (1927) who described two species. Wolcott (1948) reported seven genera and Wray (1953) included nine additional genera to the list. Mari Mutt (1976), Mari Mutt (1977), Mari Mutt (1979), Mari Mutt (1981), Mari Mutt (1982), Mari Mutt (1984), Mari Mutt (1985a), Mari Mutt (1985b), Mari Mutt (1986b), Mari Mutt (1987), Mari Mutt (1988), Mari Mutt (1986a) reported more than 60 species and described 27 new species from the Island. Soto-Adames (1988a), Soto-Adames (1988b), Soto-Adames (2002a) reported 39 from the US Virgin Islands and Puerto Rico and described nine species. Samalot-Roque (2006) found 44 species in red mangrove forest (*Rhizophoramangle*) around the Island. Ospina-Sánchez (2011) found 35 species in a secondary forest in Mayagüez. Soto-Adames (unpublished data) identified 33 and Ospina-Sánchez (2019) identified 53 species from the Luquillo Experimental Forest.

The present work updates the list of Collembola of Puerto Rico published by Mari Mutt (1982), where 39 genera and 59 species were listed, including their geographic distribution according to biogeographic zones in the world. In the present revision for Collembola from Puerto Rico, we include a list of species, historical reports and world and local distribution.

## Materials and methods

**Figure 1.**
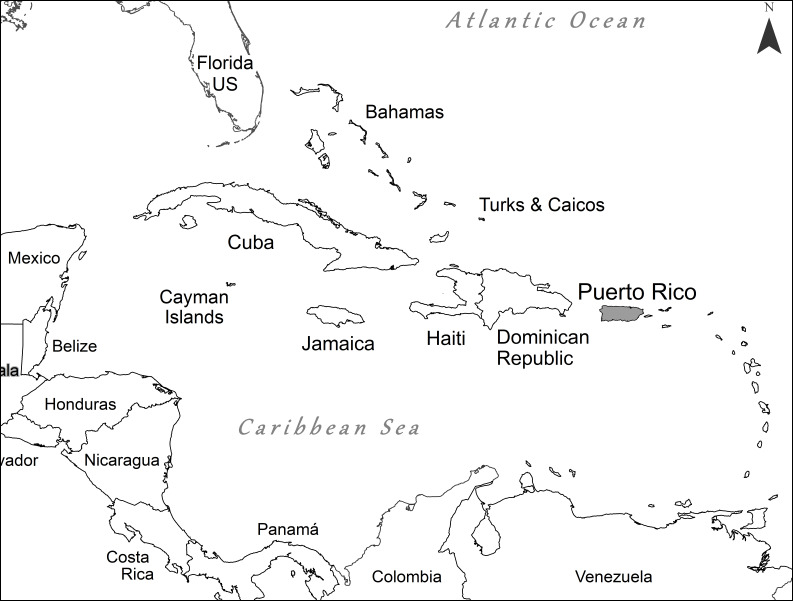
Localisation of Puerto Rico in the great Caribbean Region.

The list of species recorded from Puerto Rico (Fig. 1) is based on bibliographic references (Folsom 1927, Wolcott 1948, Wray 1953, Mari Mutt 1976, Mari Mutt 1977, Mari Mutt 1979, Mari Mutt 1981, Mari Mutt 1982, Mari Mutt 1984, Mari Mutt 1985a, Mari Mutt 1985b, Mari Mutt 1986b, Mari Mutt 1987, Mari Mutt 1988, Soto-Adames 1988a, Soto-Adames 1988b, Soto-Adames 2002a), including species reported in two unpublished master theses reported here as new records for the fauna (Samalot-Roque 2006, Ospina-Sánchez 2011) and taxonomic identifications of altitudinal gradient experiments in the Luquillo Experimental Forest (Richardson et al. 2005, Ospina-Sánchez 2019). The collection location information for each species was obtained from the cited references, when available. The data for global distribution of species is based on Bellinger et al. (2019).

Biogeographical distribution regions according to Good (1974), modified by Christiansen and Bellinger (1995) and Culik and Zeppelini (2003), were summarised for each species as follows: Boreal, Neotropical, South African, Paleotropical, Australian and Antarctic. Species distributed in four or more major regions are considered cosmopolitan (Abrantes et al. 2010, Mari Mutt 1982). The species distribution was extracted from Bellinger et al. (2019). The species reported exclusively from the Puerto Rico Bank were listed as Endemic. Nomenclatural organisation follows that of Bellinger et al. 2019.

The fields given in the online database https://doi.org/10.2737/RDS-2018-0063 are: Family, genus, species, world distribution and Neotropical distribution, historical reports in Puerto Rico, life zone in P.R, location (municipality) and habitat (Ospina-Sanchéz et al. 2019). The list of the Collembola from Puerto Rico are presented here, include: Family, genus, species, world and local distribution and historical records.

## Checklists

### Checklist of Collembola from Puerto Rico

#### Hypogastruridae

0408B694-E0BB-5ED8-807F-C9D7CAB23E8B

#### Ceratophysella denticulata

(Bagnall, 1941)

F5B1C268-F9FD-528E-A482-7923799CB13E

##### Distribution

Cosmopolitan; Puerto Rico.

##### Notes

Reported by Mari Mutt 1982 as *Hypogastruradenticulata*.

#### Microgastrura sp. "nov."

463EF932-F3DE-5BAD-814D-B5B71EDE5264

##### Distribution

Endemic; Puerto Rico: Luquillo.

##### Notes

Reported by Ospina-Sánchez 2019, new record.

#### Paraxenylla affiniformis

(Stach, 1930)

69B65246-77A8-54FE-B4BB-05398B987F73

##### Distribution

Cosmopolitan; Puerto Rico: Ceiba, Guayama, Naguabo, Santa Isabel.

##### Notes

Reported by Samalot-Roque 2006, new record.

#### Xenylla grisea

Axelson, 1900

9A965CD5-5787-58C8-9DD9-6194CD65F551

##### Distribution

Boreal, Neotropical, Austral; Puerto Rico.

##### Notes

Reported by Heatwole and Levins 1973.

#### Xenylla malayana

Salmon, 1951

CC1240DF-6DBB-5BD5-A3BA-39A6B55D2A5F

##### Distribution

Neotropical, Paleartic; Puerto Rico: Mayagüez.

##### Notes

Reported by Samalot-Roque 2006, new record.

#### Xenylla portoricensis

da Gama, 1976

3005AB3A-83E0-5F9A-89A6-85F44B8EA8E2

##### Distribution

Endemic; Puerto Rico.

##### Notes

Reported by Da Gama 1976.

#### Xenylla welchi

Folsom, 1916

A00A99D9-E483-5E9B-9E09-EFBE2CD3C98E

##### Distribution

Cosmopolitan; Puerto Rico: San Juan.

##### Notes

Reported by Wolcott 1948, Samalot-Roque 2006.

#### Xenylla yucatana

Mills, 1938

A364FC86-2B92-5757-840E-4AC29E183242

##### Distribution

Cosmopolitan; Puerto Rico.

##### Notes

Reported by Da Gama 1976.

#### Xenylla sp. "nov. 1"

BC3B3396-0486-5A4D-8AFB-3A4DCE39344A

##### Distribution

Endemic; Puerto Rico: Luquillo.

##### Notes

Reported by Ospina-Sánchez 2019, new record.

#### Xenylla sp. "nov. 2"

154E60C5-9A6E-59B9-843A-F97F3B92C2AD

##### Distribution

Endemic; Puerto Rico: Luquillo.

##### Notes

Reported by Ospina-Sánchez 2019, new record.

#### Odontellidae

2CE45F80-AEDC-5B76-9E37-C9E64B2AF14F

#### Odontella sp. 1

Mari Mutt, 1977

481149DF-E830-5BC0-90EE-EB1A61A594A6

##### Distribution

Cosmopolitan; Puerto Rico: Cayey, Luquillo.

##### Notes

Reported by Mari Mutt 1977.

#### Superodontella cf. cornifer

Mills, 1934

91C5FE60-9BCF-5283-9C07-5B93739FC2E9

##### Distribution

Cosmopolitan; Puerto Rico: Luquillo.

##### Notes

Reported by Ospina-Sánchez 2019, new record.

#### Brachystomellidae

F45807F6-4D07-524C-AD93-FC9EF24AD709

#### Brachystomella agrosa

Wray, 1953

EA41CD0C-89D2-5EA3-9837-925F29C2BE1D

##### Distribution

Neotropical; Puerto Rico: Luquillo, Mayagüez.

##### Notes

Reported by Wray 1953, Samalot-Roque 2006, Ospina-Sánchez 2011, Soto-Adames (unpublished data).

#### Brachystomella baconaensis

Gruia, 1983

8A550CC7-FBC6-5D2F-B878-469A6678D805

##### Distribution

Neotropical; Puerto Rico: Cabo Rojo, Guánica.

##### Notes

Reported by Samalot-Roque 2006, new record.

#### Brachystomella sp.

2FC4C5CD-0BEF-5802-AE30-5638E7FCCC1F

##### Distribution

Endemic; Puerto Rico: Luquillo.

##### Notes

Reported by Soto-Adames (unpublished data), new record.

#### Brachystomella sp. "nov. 1"

FFB4A7D1-CDE1-587F-9FAB-C1677B3B2383

##### Distribution

Endemic; Puerto Rico: Luquillo.

##### Notes

Reported by Ospina-Sánchez 2019, new record.

#### Brachystomella sp. "nov. 2 "

90D01416-E82A-5E01-BE50-2FD73C68EADB

##### Distribution

Endemic; Puerto Rico: Luquillo.

##### Notes

Reported by Ospina-Sánchez 2019, new record.

#### Neanuridae

7DA24243-48B6-5919-A929-B3CAC3C757FF

#### Friesea sp.

Mari Mutt, 1976

F1559C6C-8C74-5D5A-956B-78D34A064893

##### Distribution

Cosmopolitan; Puerto Rico.

##### Notes

Reported by Mari Mutt 1976.

#### Friesea josei

Palacios-Vargas, 1986

0C756A26-BDFD-5539-8F6C-1F90FC9C2D82

##### Distribution

Neotropical; Puerto Rico.

##### Notes

Reported by Palacios-Vargas 1986.

#### Friesea magnicornis

Denis, 1931

595A475A-13AC-5F62-B5DB-1B752510A615

##### Distribution

Neotropical; Puerto Rico: Luquillo.

##### Notes

Reported by Palacios-Vargas 1986.

#### Americanura interrogator

Cassagnau & Palacios-Vargas, 1983

3D90DC4E-20E7-54A9-BA68-D5B352BD9758

##### Distribution

Neotropical; Puerto Rico: Mayagüez.

##### Notes

Reported by Ospina-Sánchez 2011, new record.

#### Arlesia albipes

Folsom, 1927

090D350F-3E43-55A9-AF39-40323E447270

##### Distribution

Neotropical; Puerto Rico: Mayagüez.

##### Notes

Reported by Wray 1953 as *Portachorutesmambatus*, Samalot-Roque 2006, Ospina-Sánchez 2011.

#### Arlesia sp.

783D260F-4765-52F3-A698-6BDA7BA28F9D

##### Distribution

Endemic; Puerto Rico: Luquillo.

##### Notes

Reported by Soto-Adames (unpublished data), new record.

#### Arlesia sp. "nov. "

B2C74B9E-41DA-5B18-99D6-D1EBC079D127

##### Distribution

Endemic; Puerto Rico: Luquillo.

##### Notes

Reported by Ospina-Sánchez 2019, new record.

#### Neotropiella silvestri

(Denis, 1929)

6160F4D0-5711-5A44-9B8E-D2BED7E51FC0

##### Distribution

Neotropical; Puerto Rico: Cayey, Luquillo, Maricao, Mayagüez.

##### Notes

Reported by Mari Mutt 1977, Ospina-Sánchez 2011.

#### Neotropiella sp.

08A0F850-F2DA-5D87-95BB-D70B8352FCCF

##### Distribution

Neotropical; Puerto Rico: Luquillo.

##### Notes

Reported by Soto-Adames (unpublished data), new record.

#### Furculanurida bistribusata

Ospina-Sánchez, Palacios-Vargas & González, 2020

B322BCB7-740A-54F0-BC31-6D19325E2047

##### Distribution

Endemic; Puerto Rico: Luquillo.

##### Notes

Reported by Ospina-Sánchez 2019, Ospina-Sánchez et al. 2020.

#### Pronura sp. "nov."

ADA789EC-18D3-5D5C-A2C1-D34126C1007F

##### Distribution

Endemic; Puerto Rico: Luquillo.

##### Notes

Reported by Ospina-Sánchez 2019, new record.

#### Hylaeanura infima

Arlé, 1966

E0CEC7CE-3D79-546A-A142-895111CA48CF

##### Distribution

Neotropical; Puerto Rico: Luquillo.

##### Notes

Reported by Ospina-Sánchez 2019, new record.

#### Hylaeanura sp. "nov."

122F3D1E-8FC3-5E1C-8FDB-BDE6CB958A09

##### Distribution

Endemic; Puerto Rico: Luquillo.

##### Notes

Reported by Ospina-Sánchez 2019, new record.

#### Micranurida sp. caribena

FC9BB18E-8643-5671-9911-565CB591FDC5

##### Distribution

Neotropical; Puerto Rico: Luquillo.

##### Notes

Reported by Ospina-Sánchez 2019, as a new record "*Micranuridawladimiri* n. subsp. *caribeña*".

#### Pseudachorutes parvulus

Börner, 1901

5D2E0733-D196-550C-8849-5E631E11CCEB

##### Distribution

Neotropical, Paleartic; Puerto Rico: Mayagüez.

##### Notes

Reported by Samalot-Roque 2006, Ospina-Sánchez 2011, new record.

#### Pseudachorutes sp.

2D3B61D5-24EE-52B9-9BBB-A0CDAF228C88

##### Distribution

Endemic; Puerto Rico: Luquillo.

##### Notes

Reported by Soto-Adames (unpublished data), new record.

#### Pseudachorutes sp. "nov. 1"

9A50A179-3F46-5ADD-9354-DF2538C31FD1

##### Distribution

Endemic; Puerto Rico: Luquillo.

##### Notes

Reported by Ospina-Sánchez 2019, new record.

#### Pseudachorutes sp. "nov. 2"

676D0AED-AAA1-5DEA-8F18-11B1B5FEFE7A

##### Distribution

Endemic; Puerto Rico: Luquillo.

##### Notes

Reported by Ospina-Sánchez 2019, new record.

#### Pseudanurida sawayana

Schuster, 1965

D5ECE55F-066C-550C-8DA3-0B85885D7C43

##### Distribution

Neotropical, Paleartic; Puerto Rico: Aguadilla.

##### Notes

Reported by Christiansen and Bellinger 1988, Samalot-Roque 2006.

#### Paranura sp.

Mari Mutt, 1977

0FDCDAD6-ACB7-57C3-8364-1841F8BAAFE1

##### Distribution

Holartic, Paleartic, Neotropical; Puerto Rico: Cayey, Guajacata, Luquillo, Quebradillas, San Sebastián.

##### Notes

Reported by Mari Mutt 1977, Soto-Adames (unpublished data).

#### Paranura nr. anops

Christiansen & Bellinger, 1980

19DD7A85-29F2-5D52-B3DF-B0092A1AEF94

##### Distribution

Paleartic; Puerto Rico.

##### Notes

Reported by Mari Mutt 1982.

#### Paranura nr. quadrilobata

Hammer, 1953

3DFECF84-AEE1-5C75-AE73-FF9B17CB1B6E

##### Distribution

Holartic; Puerto Rico.

##### Notes

Reported by Mari Mutt 1977.

#### Paleonura borincana

Palacios-Vargas & Soto-Adames, 2017

D2B1FF23-FE04-56BE-924A-D338D70082B0

##### Distribution

Endemic; Puerto Rico: Luquillo, Maricao.

##### Notes

Reported by Palacios-Vargas and Soto-Adames 2017.

#### Sensillanura nr. illina

(Christiansen & Bellinger, 1980)

BD1A7B1B-B4CA-503F-A3F1-131284F3BD8F

##### Distribution

Nearctic; Puerto Rico.

##### Notes

Reported by Mari Mutt 1982 as Neanuranr.illina.

#### Sensillanura sp.

D9D2A23D-5087-5C46-BA82-044B367B7091

##### Notes

Reported by Soto-Adames (unpublished data), new record.

#### Onychiuridae

230318CA-74C2-5F31-A01F-CB80FCE8BF0D

#### Onychiurus cunhai

(Arlé, 1970)

085B9CD5-15DA-5048-9443-F3F57B2C7236

##### Distribution

Neotropical; Puerto Rico: Arecibo.

##### Notes

Reported by Samalot-Roque 2006, new record.

#### Onychiurus subcadaverinus

(Denis, 1931)

894D8FA6-2A6E-58EB-8C90-973C40D9128D

##### Distribution

Neotropical; Puerto Rico: Maricao, Mayagüez.

##### Notes

Reported by Mari Mutt 1977 as *Onychiurusfimetarius*.

#### Onychiurus sp.

B73C3AEB-13BD-5561-8B84-19B0BA4D5595

##### Distribution

Paleartic; Puerto Rico: Luquillo, Mayagüez.

##### Notes

Reported by Ospina-Sánchez 2011, Soto-Adames (unpublished data), new record.

#### Thalassaphorura nr. encarpata

(Denis, 1931)

768497E7-8FF5-50EA-897A-456AA9871064

##### Distribution

Cosmopolitan; Puerto Rico: Luquillo.

##### Notes

Reported by Mari Mutt 1982 as Protaphoruranr.encarpata.

#### Thalassaphorura sp. "nov."

A52BD976-F634-566E-B145-7EFA375821A3

##### Distribution

Endemic; Puerto Rico: Luquillo.

##### Notes

Reported by Ospina-Sánchez 2019, new record.

#### Protaphorura nr. hera

(Christiansen & Bellinger, 1980)

24C341C5-F466-5498-BCFC-6411DB9451C0

##### Distribution

Cosmopolitan; Puerto Rico

##### Notes

Reported by Palacios Vargas and Díaz 1995 as Protaphoruranr.hera.

#### Tullbergiidae

FCF93DAA-D48F-5A9D-B067-FE848EFE929C

#### Tullbergia sp.

13217400-65BE-5FD5-8890-107C724834EF

##### Distribution

Cosmopolitan; Puerto Rico.

##### Notes

Reported by Mari Mutt 1982.

#### Mesaphorura yosiii

(Rusek, 1967)

882C2E59-C3D0-50C6-A03B-324AABCADD03

##### Distribution

Cosmopolitan; Puerto Rico: Mayagüez.

##### Notes

Reported by Ospina-Sánchez 2011, new record.

#### Mesaphorura cf. ruseki

Christiansen & Bellinger, 1980

03BBF93C-F328-5279-A9A0-D4285491BC63

##### Distribution

Cosmopolitan; Puerto Rico: Luquillo.

##### Notes

Reported by Ospina-Sánchez 2019, new record.

#### Isotomidae

785A7C24-024F-500D-8072-7ECE937742CF

#### Archisotoma gourbaultae

Thibaud, 1993

77CCCAD6-F335-546A-9202-615C133A19A9

##### Distribution

Neotropical; Puerto Rico.

##### Notes

Reported by Christiansen and Bellinger 1988 as *A.interstitialis*, Samalot-Roque 2006.

#### Archisotoma interstitialis

Delamare Deboutteville, 1953

B5F84ADF-FDC3-5F7E-B082-F08EA8B911AD

##### Distribution

Cosmopolitan; Puerto Rico.

##### Notes

Reported by Christiansen and Bellinger 1988.

#### Axelsonia tubifera

Strenzke, 1958

3E11B8E4-DD56-55C1-AB0A-CC2A54BEF9D9

##### Distribution

Neotropical; Puerto Rico: Guánica, Guayama, Lajas, Patillas, Salinas, Santa Isabel.

##### Notes

Reported by Samalot-Roque 2006, new record.

#### Hemisotoma thermophila

(Axelson, 1900)

98F8CC9C-61A9-5F8C-9D70-4694DBA5DC96

##### Distribution

Cosmopolitan; Puerto Rico: Aguadilla, Arecibo, Cabo Rojo, Cayey, Ceiba, Guayama, Las Marias, Luquillo, Maricao, Mayagüez, Morovis, Río Grande, Toa Baja.

##### Notes

Reported by Mari Mutt 1977 as C*ryptophygus thermophilus*, Samalot-Roque 2006, Ospina-Sánchez 2011.

#### Proisotoma sp. 1

4A90534C-10DE-5FB3-B01C-07F8F024791C

##### Distribution

Cosmopolitan; Puerto Rico.

##### Notes

Reported by Mari Mutt 1976.

#### Folsomina onychiurina

Denis, 1931

F1903C0F-48B6-5A01-8BEA-3A40A9A5758E

##### Distribution

Cosmopolitan; Puerto Rico: Mayagüez.

##### Notes

Reported by Ospina-Sánchez 2011, new record.

#### Folsomia candida

Willem, 1902

652602E1-D413-5E69-8E3E-418D64219334

##### Distribution

Cosmopolitan; Puerto Rico.

##### Notes

Reported by Peck 1974, Mari Mutt 1982.

#### Folsomia sylvia

Wray, 1953

40C922C2-9A0A-56CF-A6D4-25E97E0BC024

##### Distribution

Neotropical; Puerto Rico.

##### Notes

Reported by Wray 1953.

#### Folsomides americanus

Denis, 1931

76A79071-00D5-594C-9745-FEDCE126E6F4

##### Distribution

Tropical; Puerto Rico: Luquillo.

##### Notes

Reported by Soto-Adames (unpublished data), new record.

#### Folsomides centralis

(Denis, 1931)

DAD521DF-A02D-54A9-AD32-9A0B658B6404

##### Distribution

Neotropical, Austral, Paleartic; Puerto Rico: Arecibo, Cabo Rojo, Humacao, Mayagüez, Toa Baja.

##### Notes

Reported by Samalot-Roque 2006, Ospina-Sánchez 2011, new record.

#### Folsomides parvulus

Stach, 1922

9CC963BD-4350-5809-9BC6-44123532B32E

##### Distribution

Cosmopolitan; Puerto Rico: Arecibo, Mayagüez, Moca, San Juan, Toa Baja.

##### Notes

Reported by Mari Mutt 1977 as *Folsomidesamericanus*, Mari Mutt 1982, Samalot-Roque 2006, Ospina-Sánchez 2011.

#### Isotomodes sp.

212E7DC7-5626-5EDD-9F07-E1D05DE8C810

##### Distribution

Cosmopolitan; Puerto Rico: Mayagüez.

##### Notes

Reported by Mari Mutt 1982, Ospina-Sánchez 2011.

#### Isotomiella minor

(Schäffer, 1896)

F10C6E14-A0D8-5163-933C-E30FBEDEA85F

##### Distribution

Cosmopolitan; Puerto Rico: Arecibo, Cabo Rojo, Humacao, Luquillo, Mayagüez, San Juan Toa Baja.

##### Notes

Reported by Wray 1953, Samalot-Roque 2006, Ospina-Sánchez 2011, Soto-Adames 2005 (unpublished data).

#### Isotomiella sp.

B7259822-08D6-56E0-A68B-5076420C6890

##### Notes

Reported by Ospina-Sánchez 2019, new record.

#### Isotomurus sp. 1

82CC5674-D182-5F6D-B535-FD88BCE03159

##### Distribution

Cosmopolitan; Puerto Rico: Luquillo, Mayagüez.

##### Notes

Reported by Mari Mutt 1976, Ospina-Sánchez 2011, Soto-Adames, 2005 (unpublished data).

#### Isotomurus sp. 2

37F108A1-337C-5CE9-8E08-1CB3254AD2EF

##### Distribution

Endemic; Puerto Rico: Luquillo.

##### Notes

Reported by Ospina-Sánchez 2019, new record.

#### Psammisotoma dispar

(Christiansen & Bellinger, 1988)

8B0DBC24-6948-5B6C-8D66-87F26691FBCD

##### Distribution

Neotropical; Puerto Rico: Cabo Rojo, Ceiba, Toa Baja.

##### Notes

Reported by Samalot-Roque 2006, new record.

#### Actaletidae

B49FD005-0D82-5451-82E2-E65D0583BB15

#### Spinactaletes aebianus

Soto-Adames, 1988

C52CDE92-C319-550C-A4B3-343776CD0F00

##### Distribution

Endemic; Puerto Rico: Isla Mona.

##### Notes

Reported by Soto-Adames 1988b.

#### Spinactaletes bellingeri

Soto-Adames, 1988

8B87F230-FC86-5858-A5F8-159CE69BFC65

##### Distribution

Endemic; Puerto Rico: Guánica, Vieques.

##### Notes

Reported by Soto-Adames 1988b.

#### Spinactaletes calcalectoris

Soto-Adames, 1988

057C0024-D253-5F4F-A531-5570A1B3A3A5

##### Distribution

Endemic; Puerto Rico: Aguadilla, Guajataca, Isla Mona, Vega Baja.

##### Notes

Reported by Soto-Adames 1988b.

#### Spinactaletes myoptesimus

Soto-Adames, 1988

053BE56A-EDC1-5109-98B9-5AB7C5617932

##### Distribution

Endemic; Puerto Rico: Guánica, Vieques, Yabucoa.

##### Notes

Reported by Soto-Adames 1988b.

#### Orchesellidae

807F9098-E5DD-5242-8150-AC33819DAD55

#### Dicranocentrus celatus

Mari Mutt, 1985

09D57104-5442-557D-9DC0-F18E19C2246E

##### Distribution

Endemic; Puerto Rico.

##### Notes

Reported by Mari Mutt 1985a.

#### Dicranocentrus marias

Wray, 1953

977A24DA-7C74-522E-8414-54C5F3E8445D

##### Distribution

Endemic; Puerto Rico: Mayagüez.

##### Notes

Reported by Mari Mutt 1979, Ospina-Sánchez 2011.

#### Heteromurtrella puertoricensis

(Mari Mutt, 1979)

D2153588-8379-58C5-BDD5-C8457AB5B42A

##### Distribution

Endemic; Puerto Rico: Maricao, Mayagüez.

##### Notes

Reported by Mari Mutt 1979.

#### Heteromurtrella tihuiensis

(Mari Mutt, 1985)

2317468E-6AAC-51AE-AB61-D3D07A758248

##### Distribution

Endemic; Puerto Rico: Mayagüez.

##### Notes

Reported by Mari Mutt 1985b, Ospina-Sánchez 2011.

#### Heteromurus sp.

B233020F-69B1-5968-8A8F-AAEE792A0026

##### Distribution

Neotropical; Puerto Rico: Luquillo.

##### Notes

Reported by Soto-Adames (unpublished data), new record.

#### Entomobryidae

9CC835A4-03E9-5DEC-9281-8432199A522E

#### Calx sp.

C2258673-F86A-51F8-9694-91552DB570D1

##### Distribution

Neotropical, Holartic; Puerto Rico: Fajardo.

##### Notes

Reported by Samalot-Roque 2006, new record.

#### Calx cubensis

(Folsom, 1927)

B1EAC128-006A-5482-9713-A5A35E3E047E

##### Distribution

Neotropical; Puerto Rico.

##### Notes

Reported by Wolcott 1948 as *Entomobyacubensis*, Soto-Adames 2002b.

#### Entomobrya linda

Soto-Adames, 2002

77525943-BA84-56FB-8B61-82491D176DFF

##### Distribution

Neotropical; Puerto Rico: Humacao, Toa Baja.

##### Notes

Reported by Samalot-Roque 2006, new record.

#### Entomobrya longiseta

Soto-Adames, 2002

8ACF627B-28E4-5759-9AFC-60F9EC12BF5D

##### Distribution

Neotropical; Puerto Rico: Mayagüez, Luquillo, Orocovis.

##### Notes

Reported by Soto-Adames 2002b, Ospina-Sánchez 2011.

#### Entomobrya sp. "nov."

C95B178F-6FAC-5E28-9852-3D0F6E84B530

##### Distribution

Endemic; Puerto Rico: Luquillo.

##### Notes

Reported by Ospina-Sánchez 2019, new record.

#### Willowsia jacobsoni

(Börner, 1913)

3ED221CD-BB3F-5C90-8A00-3088EAB9FE9B

##### Distribution

Neotropical, Paleartic, Austral; Puerto Rico: Fajardo, Salinas.

##### Notes

Reported by Mari Mutt 1982, Mari Mutt and Gruia 1983, Samalot-Roque 2006.

#### Seira blanca

Mari Mutt, 1986

2C7BCA04-820E-5A57-A9BF-C8FC6C5A9120

##### Distribution

Endemic; Puerto Rico: Carolina, Cabo Rojo, Fajardo, Humacao, Río Grande.

##### Notes

Reported by Mari Mutt 1986b, Soto-Adames 2002b, Samalot-Roque 2006.

#### Seira brasiliana

(Arlé, 1939)

B70C55FF-0337-5757-94D1-B388C73707F9

##### Distribution

Neotropical, Holartic; Puerto Rico: Cabo Rojo, Carolina, Ceiba, Guayama, Peñuelas, Salinas, San Juan, Santa Isabel.

##### Notes

Reported by Mari Mutt 1986b, Samalot-Roque 2006.

#### Seira desapercibida

Soto-Adames, 2002

8E9A3A7D-7A87-5226-AC4E-61C02AA44746

##### Distribution

Endemic; Puerto Rico.

##### Notes

Reported by Soto-Adames 2002b.

#### Seira dowlingi

(Wray, 1953)

CB81DD41-BFF0-583B-A985-1785ACBC376A

##### Distribution

Neotropical, Holartic; Puerto Rico: Guánica, Luquillo.

##### Notes

Reported by Wray 1953, Mari Mutt 1986b, Samalot-Roque 2006.

#### Seira steinmetzi

(Wray, 1953)

1C7C0ABB-EF74-5A82-9FDD-D9DFF2B91D8B

##### Distribution

Neotropical, Holartic; Puerto Rico: Bayamón, Guánica, Mayagüez.

##### Notes

Reported by Mari Mutt 1986b as *Seiradistincta*.

#### Lepidocyrtus caprilesi

Wray, 1953

BA4518C3-F83D-599F-A32F-0DCA21A7C29D

##### Distribution

Neotropical; Puerto Rico: Arecibo, Cayey, Guayama, Isabela, Las Marias, Luquillo, Manatí, Maricao, Mayagüez, Moca, Orocovis, Quebradillas, Río Grande, Utuado.

##### Notes

Reported by Wray 1953, Mari Mutt 1986a, Soto-Adames 2002a, Ospina-Sánchez 2011, Ospina-Sánchez 2019.

#### Lepidocyrtus biphasis

Mari Mutt, 1986

3C992F7F-3103-5F43-93C2-9C003333EBFB

##### Distribution

Endemic; Puerto Rico: Aibonito, Aguadilla, Arecibo, Cabo Rojo, Cayey, Coamo, Guánica, Manatí, Moca, Utuado, Villalba.

##### Notes

Reported by Mari Mutt 1986a, Soto-Adames 2002a, Samalot-Roque 2006.

#### Lepidocyrtus diminutus

Mari Mutt, 1986

CF6BEB03-DC06-52EE-A937-E03EA8B46011

##### Distribution

Endemic; Puerto Rico: Cabo Rojo, Guánica, Mayagüez, Quebradillas.

##### Notes

Reported by Mari Mutt 1986a.

#### Lepidocyrtus dispar

Mari Mutt, 1986

C8527B1F-3D17-5641-9333-6AD4D2DBC253

##### Distribution

Endemic; Puerto Rico: Aguadilla, Cayey, Isabela, Luquillo, Manatí, Maricao, Mayagüez, Moca, Quebradillas, Río Grande, Utuado.

##### Notes

Reported by Mari Mutt 1986a, Soto-Adames 2002a, Ospina-Sánchez 2011.

#### Lepidocyrtus distintus

Mari Mutt, 1986

D92F7C42-5918-546D-983F-CCA9DAD023F2

##### Distribution

Endemic; Puerto Rico: Mayagüez.

##### Notes

Reported by Mari Mutt 1986a, Soto-Adames 2002a, Ospina-Sánchez 2011.

#### Lepidocyrtus finicolus

Mari Mutt, 1988

1B99E82E-D5CD-560B-9BE3-6EAF5C2B7333

##### Distribution

Endemic; Puerto Rico: Aguadilla, Vieques.

##### Notes

Reported by Mari Mutt 1988, Soto-Adames 2002a.

#### Lepidocyrtus griseolus

Mari Mutt, 1986

72020E7B-4114-5EC7-B95A-4E3DECB93039

##### Distribution

Endemic; Puerto Rico: Guánica, Mayagüez, Utuado.

##### Notes

Reported by Mari Mutt 1986a, Soto-Adames 2002a, Ospina-Sánchez 2011.

#### Lepidocyrtus ianthinus

Mari Mutt, 1986

9E9D90AE-CA0F-546F-B56B-9B3DEE8BDC7D

##### Distribution

Neotropical; Puerto Rico: Cayey.

##### Notes

Reported by Mari Mutt 1986a, Soto-Adames 2002a.

#### Lepidocyrtus lepargus

Mari Mutt, 1986

73218575-4317-58ED-A5C8-1BAD262565BC

##### Distribution

Endemic; Puerto Rico: Cabo Rojo, Guánica, Toa Baja.

##### Notes

Reported by Mari Mutt 1986a, Soto-Adames 2002a, Samalot-Roque 2006.

#### Lepidocyrtus maldonadoi

Mari Mutt, 1986

6D7C5BB2-346A-5444-9DFF-798F86C1BDE7

##### Distribution

Neotropical; Puerto Rico: Carite, Luquillo, Orocovis, Salinas, Villalba.

##### Notes

Reported by Mari Mutt 1986a, Soto-Adames 2002a.

#### Lepidocyrtus nigrosetosus

Folsom, 1927

11878589-B841-5616-9FF0-B90A24BB273D

##### Distribution

Neotropical; Puerto Rico: Adjuntas, Aguadilla, Arecibo, Barranquitas, Cabo Rojo, Caguas, Cayey, Ceiba, Coamo, Fajardo, Guayama, Humacao, Isabela, Manatí, Mayagüez, Morovis, Orocovis, Río Grande, Salinas, San Juan, San Sebastián, Toa Baja, Vega Baja.

##### Notes

Reported by Folsom 1927, Mari Mutt 1986a, Soto-Adames 2002a, Samalot-Roque 2006, Ospina-Sánchez 2011.

#### Lepidocyrtus paracaprilesi

Mari Mutt, 1988

F8DEFCE9-1F49-53B7-B64A-1D577BF04EBA

##### Distribution

Endemic; Puerto Rico: Luquillo.

##### Notes

Reported by Mari Mutt 1988, Soto-Adames 2002a, Ospina-Sánchez 2019.

#### Lepidocyrtus ramosi

Mari Mutt, 1988

E66E8964-C44C-5C1A-9E8C-66BFCC246489

##### Distribution

Endemic; Puerto Rico: Aguadilla, Añasco, Arecibo, Caguas.

##### Notes

Reported by Mari Mutt 1988, Soto-Adames 2002a.

#### Lepidocyrtus vireticulus

Mari Mutt, 1986

E2C25D8C-AE3B-518A-B023-BEC2E7BB91AD

##### Distribution

Endemic; Puerto Rico: Aguadilla, Mayagüez, San Sebastián.

##### Notes

Reported by Mari Mutt 1986a, Soto-Adames 2002a.

#### Pseudosinella biunguiculata

Ellis, 1967

C29003EE-9B9B-58FE-83FB-E51CC2E535E2

##### Distribution

Neotropical; Puerto Rico: Aguadilla, Caguas, Manatí, Mayagüez, Utuado, Villalba.

##### Notes

Reported by Mari Mutt 1982, Mari Mutt 1986a, Soto-Adames 2002a, Samalot-Roque 2006, Ospina-Sánchez 2011.

#### Pseudosinella lahainaensis

Christiansen & Luther, 1986

455CE984-03F2-5329-8ABF-D1D6173C4DF8

##### Distribution

Neotropical; Puerto Rico: Arecibo.

##### Notes

Reported by Samalot-Roque 2006, new record.

#### Pseudosinella violeta (violets)

Mari Mutt, 1986

8CC0BDD2-8D89-55F6-B64E-3D74CB048B0D

##### Distribution

Endemic; Puerto Rico: Arecibo, Cayey, Ceiba, Guayama, Isabela, Las Marias, Luquillo, Mayagüez, Orocovis, Río Grande, Toa Baja.

##### Notes

Reported by Mari Mutt 1986a, Soto-Adames 2002a, Samalot-Roque 2006, Ospina-Sánchez 2019, Soto-Adames, 2005 (unpublished data).

#### Sulcuncus borincana

Mari Mutt & Gruia, 1983

1994087A-15DC-5C3B-9444-66AB12BF09B4

##### Distribution

Endemic; Puerto Rico.

##### Notes

Reported by Mari Mutt and Gruia 1983.

#### Sulcuncus coralia

Mari Mutt & Gruia, 1983

1154D03F-3AD9-58BE-B13B-34F7F8B67AF0

##### Distribution

Endemic; Puerto Rico.

##### Notes

Reported by Mari Mutt and Gruia 1983.

#### Sulcuncus rapoporti

Massoud & Gruia, 1973

973358F7-82A1-52A2-8FF7-08084B3F6B08

##### Distribution

Endemic; Puerto Rico.

##### Notes

Reported by Mari Mutt 1982 as *Metasinellarapoporti*, Mari Mutt and Gruia 1983.

#### Sulcuncus topotypica

Bonet, 1944

C6941A76-07C7-58DC-A198-B08299625031

##### Distribution

Endemic; Puerto Rico.

##### Notes

Reported by Mari Mutt 1977 as *Metasinellatopotyca*.

#### Sulcuncus subfusa

(Wray, 1953)

FE22CF98-2D83-5EFB-B2D9-1F6ABF1A3CDD

##### Distribution

Endemic; Puerto Rico.

##### Notes

Reported by Wray 1953.

#### Paronellidae

31A3639F-D739-5D89-B212-B86A5081A77A

#### Campylothorax sabanus

(Wray,1953) Mitra, 1975

11802DDB-D91D-537E-AAB1-3F56A59D66B6

##### Distribution

Endemic; Puerto Rico: Luquillo, Mayagüez, Toa Baja.

##### Notes

Reported by Wray 1953 as *Gampylothoraxsabanus*, Mari Mutt 1987, Samalot-Roque 2006, Ospina-Sánchez 2011.

#### Trogolaphysa sp.

BFA291FE-E813-5DED-AB89-566E02054D20

##### Distribution

Neotropical, Afrotropical; Puerto Rico: Luquillo.

##### Notes

Reported by Mari Mutt 1982.

#### Trogolaphysa geminata

(Mari Mutt, 1987)

27A180B3-27E6-5B17-B5DC-70F86FA866C5

##### Distribution

Neotropical; Puerto Rico: Mayagüez.

##### Notes

Reported by Mari Mutt 1987, Ospina-Sánchez 2011.

#### Trogolaphysa jataca

(Wray, 1953)

7D6C998D-1858-5A2E-971F-37E5923753D4

##### Distribution

Neotropical; Puerto Rico: Mayagüez.

##### Notes

Reported by Wray 1953 as *Dicranocentrugajataca*, Ospina-Sánchez 2011, Ospina-Sánchez 2019.

#### Trogolaphysa luquillensis

(Mari Mutt, 1987)

27931F87-FC3E-539B-9C26-9E5589E14308

##### Distribution

Neotropical; Puerto Rico: Luquillo.

##### Notes

Reported by Mari Mutt 1987.

#### Trogolaphysa riopedrensis

(Mari Mutt, 1987)

90FA48E1-A262-5387-A2B3-D18C7A4F3643

##### Distribution

Neotropical; Puerto Rico: San Juan.

##### Notes

Reported by Mari Mutt 1987.

#### Trogolaphysa subterranea

(Mari Mutt, 1987)

4DF1CD1C-E70F-502A-9615-A2C265A62ACC

##### Distribution

Endemic; Puerto Rico.

##### Notes

Reported by Mari Mutt 1987.

#### Lepidonella marimutti

Soto-Adames & Bellini, 2015

F532DB21-7DA0-531C-9018-51AB0EDFE5D9

##### Distribution

Neotropical; Puerto Rico.

##### Notes

Reported by Mari Mutt 1987 as *Microparonellaincerta*, Soto-Adames and Bellini 2015.

#### Salina tristani

Denis, 1931

8FFF02CA-EF42-5531-B11D-DAB0BD42EDD5

##### Distribution

Neotropical; Puerto Rico: Aguadilla, Arecibo, Cabo Rojo, Luquillo, Mayagüez, Río Grande, Toa Baja.

##### Notes

Reported by Mari Mutt 1982, Mari Mutt 1987, Samalot-Roque 2006, Ospina-Sánchez 2011, Soto-Adames 2005 (unpublished data).

#### Salina wolcotti

Folsom, 1927

7344ECBC-FBD9-5694-88DA-93F704156480

##### Distribution

Endemic; Puerto Rico: Bayamón, Isabela, Peñuelas San Juan.

##### Notes

Reported by Folsom 1927, Mari Mutt 1987.

#### Cyphoderus inaequalis

Folsom, 1927

6EFBB619-61CD-57D0-9D62-86731025A8FC

##### Distribution

Neotropical; Puerto Rico: Mayagüez.

##### Notes

Reported by Wolcott 1948, Ospina-Sánchez 2011.

#### Cyphoderus similis

Folsom, 1927

21A02917-46E1-5B8B-B0BF-6337C8C24E85

##### Distribution

Paleartic, Boreal, Neotropical; Puerto Rico: Arecibo, Cabo Rojo, Ceiba, Mayagüez, Toa Baja, Vega Baja.

##### Notes

Reported by Folsom 1927, Samalot-Roque 2006.

#### Serroderus sp. "nov."

52E84939-3671-53B6-9A0F-6591EAF1C4E9

##### Distribution

Endemic; Puerto Rico: Luquillo.

##### Notes

Reported by Ospina-Sánchez 2019, Soto-Adames (unpublished data), new record.

#### Oncopoduridae

F2B7547F-E823-5E19-907E-D1102B7AB75C

#### Oncopodura arecibena

Mari Mutt, 1984

9620B819-CB6C-5CC3-80BF-C7568929958C

##### Distribution

Endemic; Puerto Rico: Arecibo, Luquillo.

##### Notes

Reported by Mari Mutt 1984, Ospina-Sánchez 2019.

#### Arrhopalitidae

D5B6E143-942E-59EF-B99D-29D5EDBA6F0B

#### Arrhopalites sp.

FB2359E8-DE42-5D96-B031-6250FA15CD0B

##### Notes

Reported by Ospina-Sánchez 2019, new record.

#### Collophora quadrioculata

Denis, 1933

CB471663-A37D-5D3D-A082-45F8CB169CBC

##### Distribution

Neotropical, Paleartic; Puerto Rico: Adjuntas, Humacao, Mayagüez, San Sebastián.

##### Notes

Reported by Mari Mutt 1977, Samalot-Roque 2006, Ospina-Sánchez 2011.

#### Dicyrtomidae

D1540D98-4928-52AF-B642-7E5350A8F464

#### Ptenothrix borincana

Soto-Adames, 1988

AC8CA1A9-547D-5669-88E3-F8401169E56F

##### Distribution

Endemic; Puerto Rico: Aibonito, Arecibo, Cayey, Las Marias, Mayagüez, Luquillo, Utuado, Villalba.

##### Notes

Reported by Soto-Adames 1988a, Ospina-Sánchez 2011, Soto-Adames (unpublished data).

#### Dicyrtoma mangle

Soto-Adames, 1988

469A153E-0013-5152-87D2-A2124A2A3DE2

##### Distribution

Endemic; Puerto Rico: Aguadilla.

##### Notes

Reported by Soto-Adames 1988a.

#### Calvatomina discolor

(Schött, 1902) Betsch, 1980

96367E68-A35D-58E9-8685-6EFD20D96E11

##### Distribution

Boreal, Neotropical, Paleartic; Puerto Rico: Aguadilla, Mayagüez.

##### Notes

Reported by Samalot-Roque 2006, new record.

#### Calvatomina nymphascopula

Soto-Adames, 1988

9D1ED20A-33A7-56C0-91EB-BEB79021617C

##### Distribution

Endemic; Puerto Rico: Aguadilla, Arecibo, Guayama, Cabo Rojo, Coamo, Mayagüez, San Juan, Toa Baja.

##### Notes

Reported by Soto-Adames 1988a, Samalot-Roque 2006.

#### Calvatomina rufescens

(Reuter, 1890) Betsch, 1980

30C09E7B-645E-54FC-B19D-B0FC857D44CF

##### Distribution

Boreal, Neotropical, Paleartic; Puerto Rico: Luquillo, Mayagüez.

##### Notes

Reported by Soto-Adames 1988a, Ospina-Sánchez 2011, Soto-Adames (unpublished data).

#### Calvatomina sp. "nov. nr. rossi"

207E5A0F-3661-5F58-8922-1E550BA04AA5

##### Distribution

Neotropical; Puerto Rico: Mayagüez, Toa Baja.

##### Notes

Reported by Samalot-Roque 2006, new record.

#### Calvatomina sp. "nov. 1"

1337902F-D6F4-5850-9EBF-B37CE45757FC

##### Distribution

Neotropical; Puerto Rico: Ceiba, Humacao, Mayagüez, Naguabo, Río Grande, San Juan, Toa Baja, Vega Baja.

##### Notes

Reported by Samalot-Roque 2006, new record.

#### Calvatomina sp. "nov. 2"

318AD05E-13B5-5E24-A467-4A4588DCCD16

##### Distribution

Neotropical; Puerto Rico: Mayagüez, Toa Baja.

##### Notes

Reported by Samalot-Roque 2006, new record.

#### Calvatomina sp. "nov. 3"

02616D44-F5F6-5CCC-8253-5F86C5D8D618

##### Distribution

Neotropical; Puerto Rico: Mayagüez.

##### Notes

Reported by Ospina-Sánchez 2011, new record.

#### Calvatomina sp. "nov. 4"

916AF289-9F35-5997-A86A-3BA6076A96B6

##### Distribution

Neotropical; Puerto Rico: Mayagüez.

##### Notes

Reported by Ospina-Sánchez 2011, new record.

#### Sminthurididae

36D37377-F3CE-51F8-9F8B-AA111C87F4F1

#### Sphaeridia sp. "nov. 1"

DADD79BD-174C-5764-9AD3-265A69C5F5D8

##### Distribution

Neotropical; Puerto Rico: Arecibo, Guayama, Lajas, Patillas.

##### Notes

Reported by Samalot-Roque 2006, new record.

#### Sphaeridia sp. 1

A062862C-DB07-5BAD-B8A4-3E442ACBE58A

##### Distribution

Neotropical; Puerto Rico: Arecibo, Vega Baja.

##### Notes

Reported by Samalot-Roque 2006, new record.

#### Sphaeridia sp. 2

1E2739F8-1155-5699-8374-35E443FF288D

##### Distribution

Neotropical; Puerto Rico: Arecibo, Guayama, Lajas, Patillas.

##### Notes

Reported by Samalot-Roque 2006, new record.

#### Sphaeridia sp. 3

B8839830-2CAD-5ADA-8017-1FD2A5A4494C

##### Distribution

Neotropical; Puerto Rico: Luquillo.

##### Notes

Reported by Soto-Adames (unpublished data), new record.

#### Sminthuridae

3F1C8FE3-C8E6-5A68-B413-FFBA202F90BC

#### Sphyrotheca aleta

Wray, 1953

982FE70B-05AF-51DD-9DDE-7D8D80700B28

##### Distribution

Endemic; Puerto Rico: Luquillo.

##### Notes

Reported by Wray 1953, Ospina-Sánchez 2019.

#### Bourletielidae

B77F5957-4950-502D-95FE-91C5D88E7A9C

#### Stenognathriopes sp. "nov."

B2B14E35-9EB3-5B90-9396-072E30F278AA

##### Distribution

Neotropical, Paleartic; Puerto Rico: Cabo Rojo, Ceiba.

##### Notes

Reported by Samalot-Roque 2006, new record.

#### Bourletiella sp.

60BBD986-5291-5E67-A243-463AF0E6D158

##### Distribution

Cosmopolitan; Puerto Rico: Isabela, Luquillo.

##### Notes

Reported by Mari Mutt 1977, Soto-Adames (unpublished data).

#### Neelidae

EA1E7B93-0277-57F4-BB66-404AFC6FF2ED

#### Neelides minutus

(Folsom, 1901) Bonet, 1947

57991AFA-C60B-5BDB-B582-814607787C0D

##### Distribution

Boreal, Neotropical, Paleartic; Puerto Rico: Luquillo.

##### Notes

Reported by Mari Mutt 1982, Soto-Adames (unpublished data).

#### Neelus desantisi

Najt, 1971

D5132ACC-2953-5044-84FD-2EF1C8A7A078

##### Distribution

Cosmopolitan; Puerto Rico: Luquillo; Puerto Rico: Luquillo.

##### Notes

Reported by Ospina-Sánchez 2019, new record.

#### Neelus murinus

Folsom, 1896

FF6AAA2E-B369-5200-80B0-4BAEA4D32D24

##### Distribution

Austral, Boreal, Neotropical, Paleartic; Puerto Rico: Luquillo, Mayagüez.

##### Notes

Reported by Ospina-Sánchez 2011, Soto-Adames (unpublished data), new record.

## Analysis

Of the 33 families of springtails recognised worldwide (Bellinger et al. (2019), 17 have been reported from Greater Puerto Rico. Amongst the major cosmopolitan families, only Tomoceridae is not represented in the islands. Most small families, not reported from Greater Puerto Rico (i.e. Poduridae, Gulgastruridae, Pachytullbergiidae, Paleotullbergiidae, Mackenziellidae and Spinothecidae), represent divergent lineages endemic to geographical regions distant from the Caribbean and are unlikely to be found here. Sturmiidae is a Neotropical family of the small order Symphypleona that typically occur on epiphytic mosses. Mosses have not been widely sampled for springtails in Greater Puerto Rico and it is possible that *Sturmius* Bretfeld, 1994 may still be found in the Islands. Isotogastruridae, Coenaletidae are known from other Antilles (e.g. *Coenaletes* has been reported from Hispaniola and St. Croix) and they might likely occur in Greater Puerto Rico, although none has been recorded yet.

**Figure 3.**
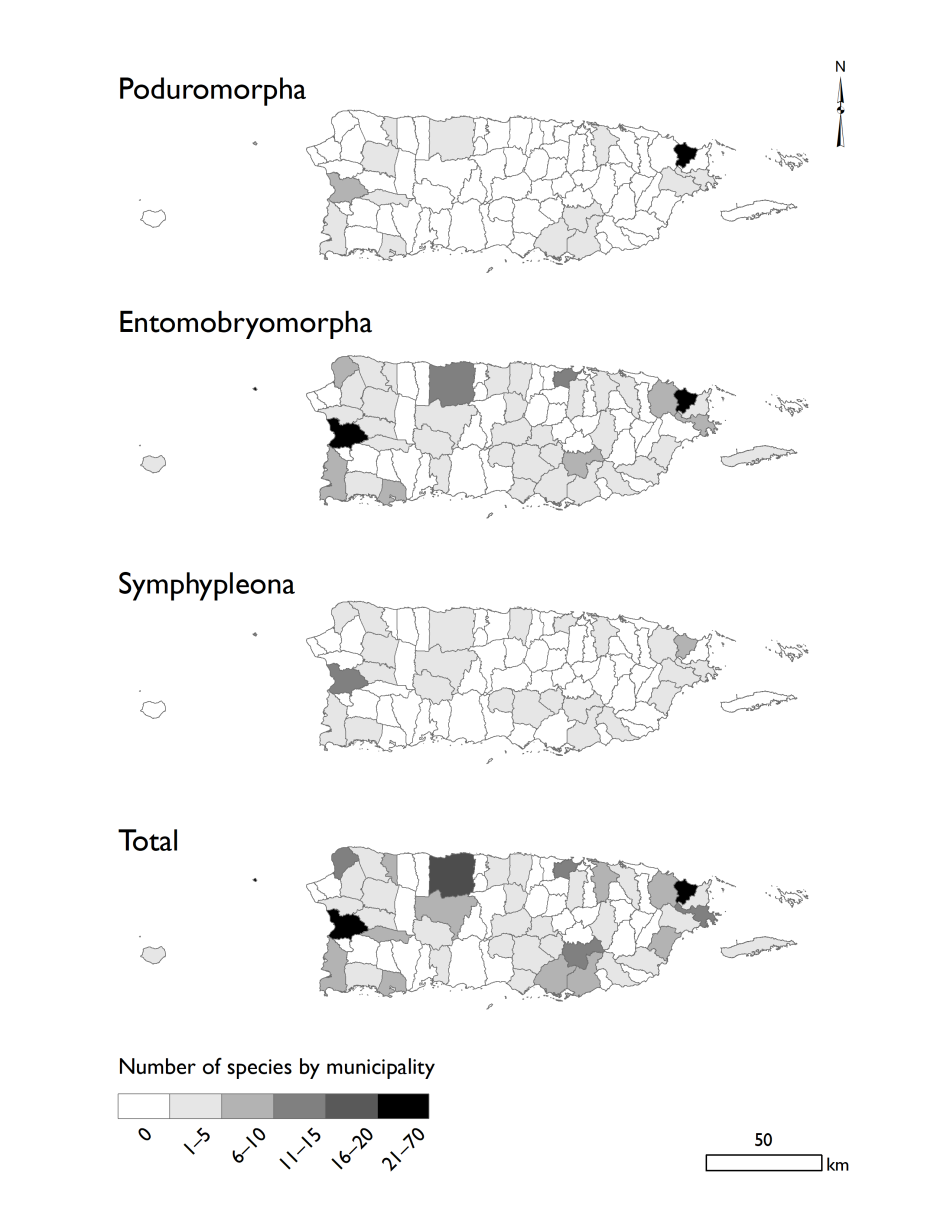
Distribution of the recorded species of Collembola in Puerto Rico, darker areas indicate higher number of species in the locality.

Politically, the Island is divided into 78 municipalities, 45 of them covered in this database (Fig. 3). According to the present inventory, the largest number of species comes from Luquillo (70 spp.), followed by Mayagüez (47 spp.) and Arecibo (17 spp., Table 1). The Collembola fauna in Puerto Rico is distributed in three principal habitats: forests, littorals and caves. In this inventory, 127 species are reported in forests (including mangrove habitats). *Archisotomagourbaultae, A.interstitialis*, *S.aebianus*, *S.bellingeri*. *S.calcalectoris* and *S.myoptesimus* are reported from littorals habitats. *Sulcuncustopotypica, O.arecibena*, *T.subterranea* and *C.quadrioculata* were reported in caves. Amongst the listed species from Puerto Rico, 58 have Neotropical distribution, 54 are Endemic and 24 are present in more than three world regions.

In the present dataset, we report that the most abundant order is Entomobryomorpha with 73 species. Amongst Entomobryomorpha, the family with the largest number of species is Entomobryidae (33); followed by Isotomidae (17). Poduromorpha with 51, the families Neanuridae (25) and Hypogastruridae (10) have the largest number of species (Table 2). In Neelipelona and Symphypleona, most families are represented by a single species, except for Dicyrtomidae. *Lepidocyrtus* Bourlet, 1839 (Entomobryidae), is the genus best represented in the islands, with 14 species, followed by *Calvatomina* Yosii, 1966 (Dicyrtomidae) represented by 8 spp. (Fig. 2, Table 2).

## Figures and Tables

**Figure 2a.**
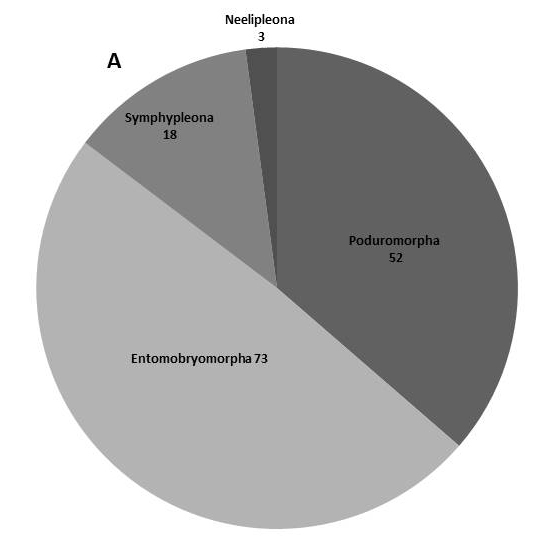
Orders of Collembola

**Figure 2b.**
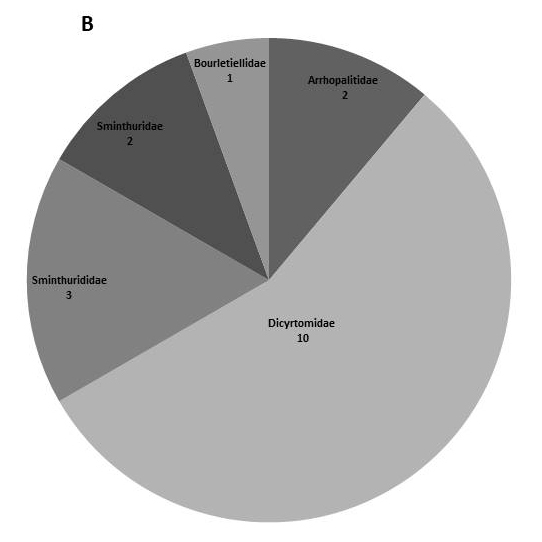
Families within the order Symphypleona

**Figure 2c.**
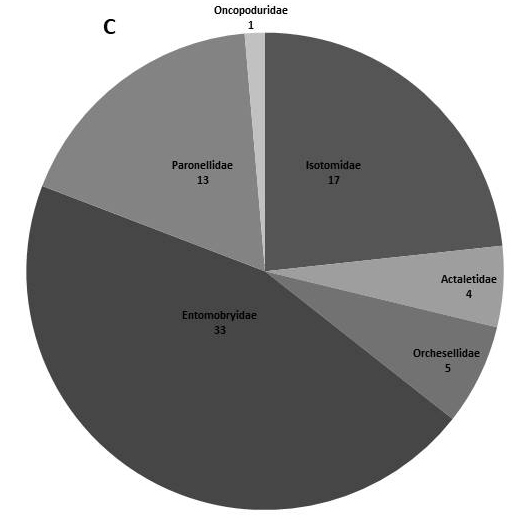
Families within the order Entomobryomorpha

**Figure 2d.**
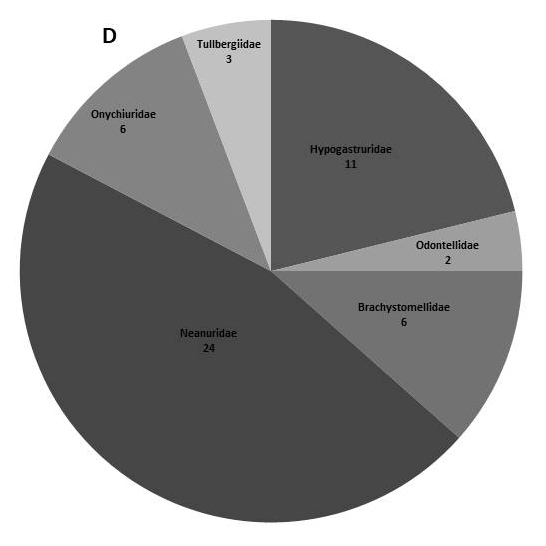
Families within the order Poduromorpha

**Table 1.** Municipalities sampled in Puerto Rico and number of Collembola species reported for each one.

**Municipality**	**No. of species**	**Municipality**	**No. of species**	**Municipality**	**No. of species**
Adjuntas	2	Guajacata	2	Orocovis	5
Aguadilla	13	Guánica	10	Patillas	2
Aibonito	2	Guayama	9	Peñuelas	2
Añasco	1	Humacao	6	Quebradillas	4
Arecibo	17	Isabela	5	Río Grande	7
Barranquitas	1	Isla Mona	3	Salinas	6
Bayamón	2	Lajas	2	San Juan	7
Cabo Rojo	9	Las Marias	4	San Sebastián	4
Caguas	3	Luquillo	70	Santa Isabel	2
Carite	1	Manatí	5	Toa Baja	15
Carolina	2	Maricao	8	Utuado	6
Cayey	11	Mayagüez	47	Vega Baja	4
Ceiba	14	Moca	4	Vieques	3
Coamo	3	Morovis	1	Villalba	4
Fajardo	3	Naguabo	2	Yabucoa	1

**Table 2.** Distribution of Collembola species in Puerto Rico.

Family	Genus	No. Genera	No. Species	No. Localities
**Hypogastruridae**		**4**	**11**	**7**
	*Ceratophysella*		1	N.I.
	*Microgastrura*		2	1
	*Paraxenylla*		1	4
	*Xenylla*		7	4
**Odontellidae**		**2**	**2**	**2**
	*Odontella*		1	1
	*Superodontella*		1	1
**Brachystomellidae**		**1**	**6**	**4**
	*Brachystomella*		5	4
	*Folsomiella*		1	1
**Neanuridae**		**12**	**24**	**8**
	*Friesea*		3	1
	*Americanura*		1	1
	*Arlesia*		3	1
	*Neotropiella*		2	4
	*Pronura*		1	1
	*Hylaeanura*		2	1
	*Micranurida*		1	1
	*Pseudachorutes*		4	3
	*Pseudanurida*		1	N.I
	*Paranura*		3	5
	*Paleonura*		1	2
	*Sensillanura*		1	N.I
**Onychiuridae**		**4**	**6**	**4**
	*Orthonychiurus*		2	3
	*Onychiurus*		1	2
	*Protaphorura*		1	N.I.
	*Thalassaporura*		2	1
**Tullbergiidae**		**2**	**3**	**2**
	*Tullbergia*		1	1
	*Mesaphorura*		2	1
**Isotomidae**		**11**	**17**	**24**
	*Archisotoma*		2	N.I
	*Axelsonia*		1	6
	*Hemisotoma*		1	13
	*Proisotoma*		1	N.I.
	*Folsomina*		1	1
	*Folsomia*		2	N.I.
	*Folsomides*		3	6
	*Isotomodes*		1	1
	*Isotomiella*		2	8
	*Isotomurus*		2	2
	*Psammisotoma*		1	3
**Actaletidae**	*Spinactaletes*	**1**	**4**	**7**
**Orchesellidae**		**3**	**5**	**3**
	*Dicranocentrus*		2	2
	*Heteromurtella*		2	3
	*Heteromurus*		1	1
**Entomobryidae**		**7**	**33**	**30**
	*Calx*		2	1
	*Entomobrya*		3	6
	*Willowsia*		1	2
	*Seira*		5	13
	*Lepidocyrtus*		14	29
	*Pseudosinella*		3	19
	*Sulcuncus*		5	N.I
**Paronellidae**		**6**	**13**	**14**
	*Campylothorax*		1	3
	*Trogolaphysa*		6	2
	*Lepidonella*		1	N.I.
	*Salina*		2	12
	*Cyphoderus*		2	7
	*Serroderus*		1	1
**Oncopoduridae**	*Oncopodura*	**1**	**1**	**2**
**Collophoridae**		**2**	**2**	**5**
	*Arrhopalites*		1	1
	*Collophora*		1	4
**Dicyrtomidae**		**3**	**10**	**17**
	*Ptenothrix*		1	9
	*Dicyrtoma*		1	1
	*Calvatomina*		8	14
**Sminthurididae**	*Sphaeridia*	**1**	**3**	**6**
**Sminthuridae**	*Sphyrotheca*	**2**	**1**	**1**
**Bourletielidae**		**2**	**2**	**3**
	*Stenognathriopes*		1	1
	*Bourletiella*		1	2
**Neelidae**		**2**	**3**	**2**
	*Neelides*		1	1
	*Neelus*		2	2
N.I. No location Information				
